# MXene Analogue: A 2D Nitridene Solid Solution for High‐Rate Hydrogen Production

**DOI:** 10.1002/anie.202203850

**Published:** 2022-05-03

**Authors:** Huanyu Jin, Huimin Yu, Haobo Li, Kenneth Davey, Taeseup Song, Ungyu Paik, Shi‐Zhang Qiao

**Affiliations:** ^1^ School of Chemical Engineering and Advanced Materials The University of Adelaide Adelaide SA 5005 Australia; ^2^ Institute for Sustainability, Energy and Resources The University of Adelaide Adelaide SA 5005 Australia; ^3^ Future Industries Institute University of South Australia Mawson Lakes Campus Adelaide SA 5095 Australia; ^4^ Department of Energy Engineering Hanyang University Seoul 04763 Republic of Korea

**Keywords:** 2D Nitridene, Catalyst Design, Electrocatalysis, Hydrogen Evolution, MXenes

## Abstract

Electrocatalysts for high‐rate hydrogen evolution reaction (HER) are crucial to clean fuel production. Nitrogen‐rich 2D transition metal nitride, designated “nitridene”, has shown promising HER performance because of its unique physical/chemical properties. However, its synthesis is hindered by the sluggish growth kinetics. Here for the first time using a catalytic molten‐salt method, we facilely synthesized a V−Mo bimetallic nitridene solid solution, V_0.2_Mo_0.8_N_1.2_, with tunable electrocatalytic property. The molten‐salt synthesis reduces the growth barrier of V_0.2_Mo_0.8_N_1.2_ and facilitates V dissolution via a monomer assembly, as confirmed by synchrotron spectroscopy and ex situ electron microscopy. Furthermore, by merging computational simulations, we confirm that the V doping leads to an optimized electronic structure for fast protons coupling to produce hydrogen. These findings offer a quantitative engineering strategy for developing analogues of MXenes for clean energy conversions.

## Graphical abstract

A new MXene analogue, V‐Mo‐based 2D layered transition metal nitride solid solution (V_0.2_Mo_0.8_N_1.2_), was synthesized via a catalytic molten‐salt method. It exhibited excellent activity and stability for hydrogen evolution reaction, superior to most MXenes. Boosted electrocatalytic performance originates from an optimized electronic structure induced by V atom alloying.

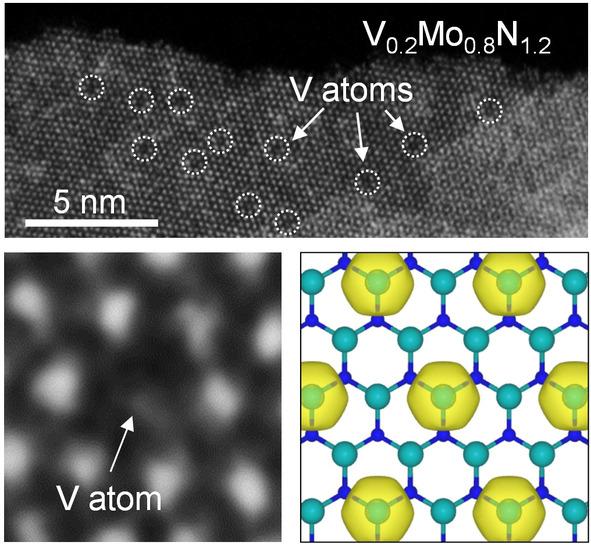

## Introduction

The production of high‐purity green hydrogen from electrocatalytic water splitting is an acknowledged path to sustainable carbon neutrality.^[1]^ The cost of electrocatalysts is a key determinant for green hydrogen.^[2]^ Most water electrolyzers use conventional noble metal‐based electrocatalysts for hydrogen evolution reaction (HER) because of the inherent high activity.^[3]^ However, high price and low reserves of these elements limit large‐scale application.^[4]^ Recently, noble metal‐free layered transition metal carbides and nitrides, known as MXenes (M_*n*+1_X_*n*_T_*x*_ M=early transition metals; X=C and/or N; T_*x*_=surface terminations), have been recognized as practically promising HER catalysts.^[5]^ However, pristine two‐dimensional (2D) MXenes have an unsatisfactory catalytic performance and inherent oxidative instability.^[5c]^ Complex post‐reaction optimizations are often needed to boost overall HER performance.^[6]^ Therefore, new and high‐rate 2D MXene‐based HER electrocatalysts are essential to the emergent green hydrogen economy.^[7]^

Nitrogen‐rich 2D layered transition metal nitride (TMN) as a MXene analogue has high electrical conductivity, corrosion resistance, and good water splitting stability.[^7d^, ^8^] We designate this material as “nitridene” because of its structural analogy with MXene. Even though nitridene does not follow the general formula M_*n*+1_X_*n*_T_*x*_, it has similar physical/chemical properties to MXene, including the 2D layered structure, high electron conductivity and surface terminations.^[9]^ However, pristine nitridene exhibits unsatisfactory HER activity because of a suboptimal hydrogen bonding energy on the atomic‐thin crystal surface.^[10]^ Satisfactory activity and corrosion resistance in nitridene are practically difficult to produce simultaneously.

Alloying is considered an “ideal” way to optimize the physical and chemical properties of 2D materials.^[11]^ Manipulating the stoichiometry of the foreign metal atoms within the lattice can tailor the matrix properties for particular applications with new functionality.^[12]^ For example, both electronic and optical properties of 2D transition metal carbide MXene have been tuned by forming solid solutions.^[13]^ The metal‐1/metal‐2 ratio can be readily controlled within the 2D crystal with alloying.^[14]^ However, alloying foreign metal atoms into a 2D TMN MXene matrix is synthetically challenging because of acknowledged sluggish growth kinetics.[^8a^, ^10^, ^15^] Additionally, etching and exfoliation are primarily impractical for many TMN‐based MXenes because of the high kinetic energy of M−N bonding.^[16]^ For example, the etching process usually destabilize the 2D lattice, leading to the formation of metal oxide instead of 2D TMNs.^[17]^ For principally these reasons, 2D TMN MXene solid solution has not been synthesized.^[17]^ Therefore, the development of novel 2D nitridiene solid solutions is significant to electrocatalysis and 2D materials chemistry.

Here for the first time, we synthesized a V−Mo bimetallic 2D nitridene solid solution, V_0.2_Mo_0.8_N_1.2_, via catalytic molten‐salt method. This synthesis ensures a facile growth of nitridene solid solution by lowering the growth barrier of metastable 2D crystals. Additionally, the gas‐liquid environment of molten salt provides a monomer assembly, facilitating a substitutional dissolution of V atoms into the V_0.2_Mo_0.8_N_1.2_ lattice. The V_0.2_Mo_0.8_N_1.2_ exhibited excellent HER activity and stability in acid with a small overpotential of 158 mV at the current density of 10 mA cm^−2^ (η_10_) together with a long‐term HER stability over 100 h. The growth mechanism for V_0.2_Mo_0.8_N_1.2_ has been elucidated via advanced synchrotron‐based spectroscopy and ex situ aberration‐corrected transmission electron microscopy. Synchrotron‐based X‐ray absorption near edge structure (XANES) and density functional theory (DFT) calculations confirmed that the boosted HER activity of the solid solution originated from the charge redistribution and unique V−N‐Mo bonding. The alloyed V atoms provide electrons to the Mo site and ensure an electron‐rich active site that promotes hydrogen coupling. We conclude that these findings offer a quantitative strategy for developing MXene analogues for high‐rate hydrogen production and will benefit the design of new 2D materials based on earth‐abundant transition metals.

## Results and Discussion

**Figure 1:**
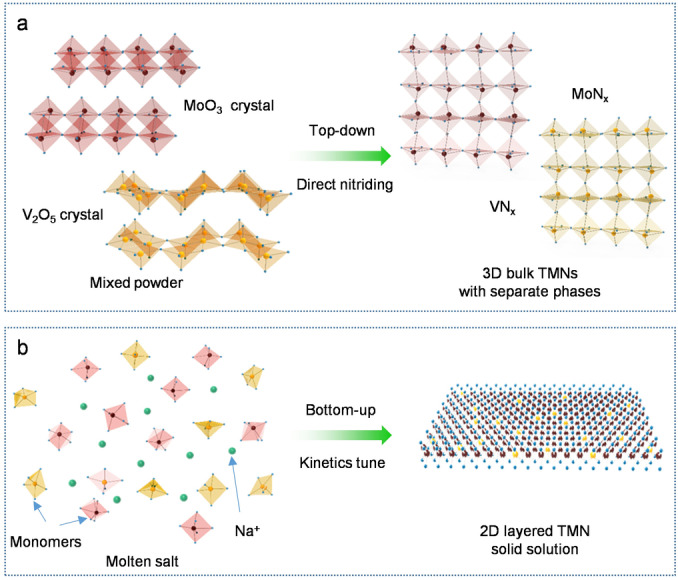
Schematic for the synthesis of a) conventional TMNs and b) 2D V_0.2_Mo_0.8_N_1.2_ solid solution through nitridation under NH_3_. Direct nitriding of transition metal oxide is a top‐down method that facilitates the formation of 3D bulk TMNs. In contrast, the molten‐salt environment melts the crystalline precursors into monomers, enabling the growth of a metastable 2D V_0.2_Mo_0.8_N_1.2_ solid solution.

The 2D layered V_0.2_Mo_0.8_N_1.2_ was synthesized via a modified molten‐salt method. Precursors of vanadium and molybdenum oxides were mixed with differing V/Mo ratios. Then the metal oxides and Na_2_MoO_4_ were blended via ball‐milling. The V_0.2_Mo_0.8_N_1.2_ solid solution was obtained by calcination of metal oxides and alkali salt mixture under NH_3_ atmosphere at 650 °C for 2 h. Note that the growth of most metal nitrides is limited by the sluggish kinetics of dissolving N atoms into the metal lattice because of the competition from the recombination of nitrogen molecules and subsequent desorption.[^15^, ^18^] Therefore, phase separation in the mixture occurs despite the two nitrides being isostructural, Figure 1a. In addition, the stable phases of metal nitrides are usually nitrogen‐deficient with 3D atomic structures that limit the diversity of 2D TMNs (Figure S1). Alternatively, the molten‐salt method can provide a liquid‐gas synthesis, significantly lowering the growth barrier of many 2D materials.[^7c^, ^19^] As is schematically shown in Figure 1b, the V_2_O_5_ and MoO_3_ are melted into [VO_5_]_pyramidal_ and [MoO_6_]_octahedron_ prior to nitridation, followed by the growth of 2D nitridene solid solution with the induction of Na ions.[^19b^, ^20^]

**Figure 2:**
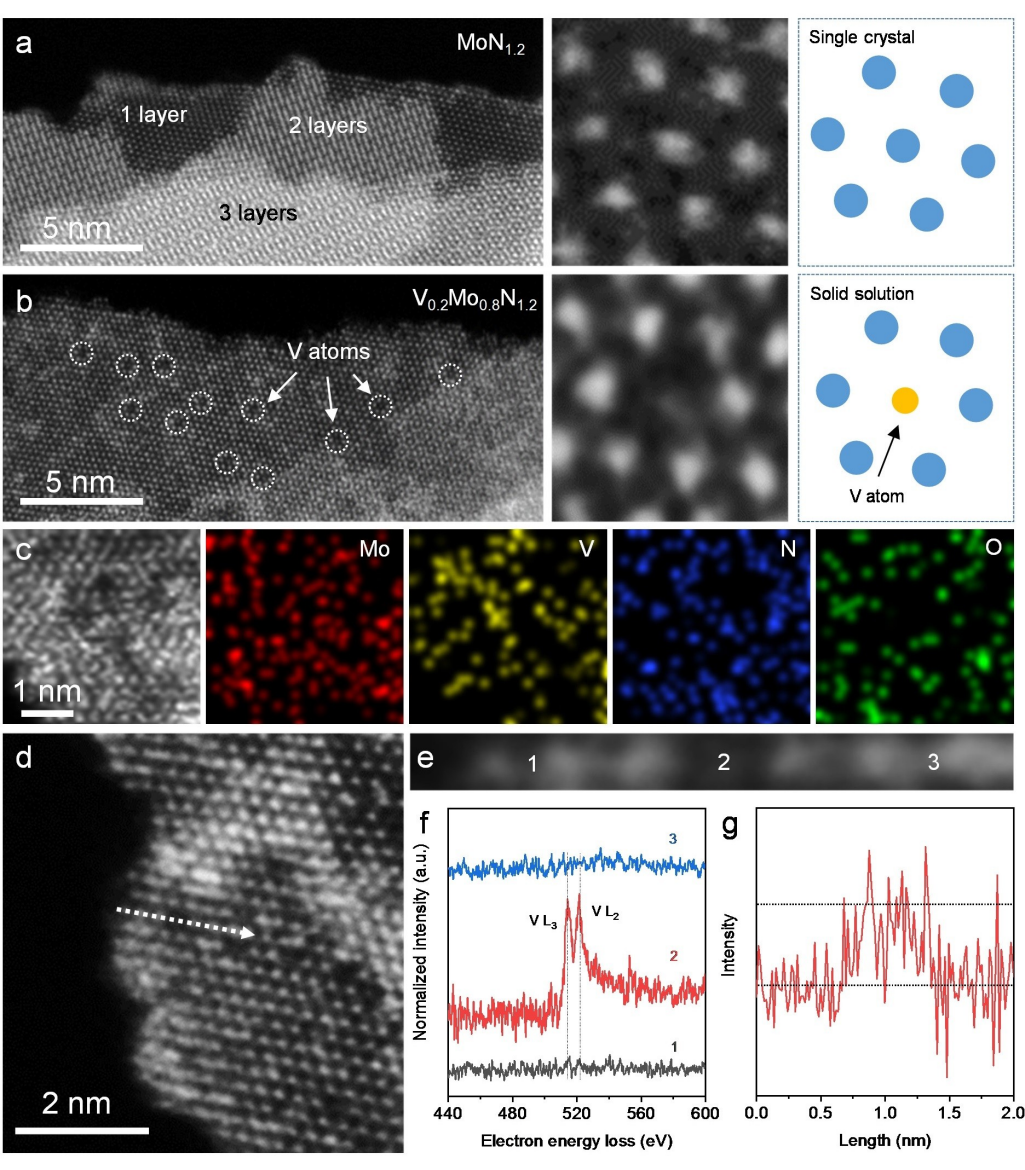
Structural analyses for V_0.2_Mo_0.8_N_1.2_ solid solution via electron microscopy. a) HAADF‐STEM image of MoN_1.2_ without V doping confirming neat hexagonal crystal lattice. b) HAADF‐STEM image of 2D V_0.2_Mo_0.8_N_1.2_ solid solution. The white‐colour dot‐circles highlight V atoms and poor contrast compared with Mo atoms. c) Elemental mapping for V_0.2_Mo_0.8_N_1.2_ confirms elements distribution in the lattice. d) and e) HAADF‐STEM image of 2D V_0.2_Mo_0.8_N_1.2_ highlighting the area of liner EELS. f) High loss EELS spectra for V_0.2_Mo_0.8_N_1.2_ corresponding to (d) and (e). g) V L edge intensity for liner EELS analyses.

The 2D V_0.2_Mo_0.8_N_1.2_ is used in this work as an example to demonstrate the crystal structure and elemental arrangement of the nitridene solid solution. The morphology of V_0.2_Mo_0.8_N_1.2_ and other control samples were characterized using scanning electron microscopy (SEM). Both MoN_1.2_ and V_0.2_Mo_0.8_N_1.2_ are 2D nanosheets with lateral sizes around 1 μm (Figure S2 and S3). Aberration‐corrected high‐angle annular dark‐field scanning transmission electron microscopy (HAADF‐STEM) was used to analyze the V atom arrangement in V_0.2_Mo_0.8_N_1.2_ at atomic scale. As a control sample, the single‐crystal MoN_1.2_ exhibited a hexagonal structure with an ordered in‐plane lattice, Figure 2a. Similarly, V_0.2_Mo_0.8_N_1.2_ exhibits hexagonal symmetry of the planes without visible lattice variation. However, significant dark sites were observed as is seen in Figure 2b, which are V atoms because of lower contrast compared with Mo under STEM mode. The nano laminated structure of the two samples can be readily seen in the cross‐sectional HAADF‐STEM images. The single‐layered thickness is about 0.7 nm (Figure S4), and the dissolved V atoms did not change the thickness of the single nanosheet (Figure S5).

The atomic‐level energy‐dispersive X‐ray spectroscopy (EDS) mapping under STEM mode highlights that the V atoms have been successfully alloyed into the MoN_1.2_ lattice, Figure 2c. The elemental distribution demonstrates the existence of O‐based terminations on the surface of V_0.2_Mo_0.8_N_1.2_. Large‐area EDS mapping of V_0.2_M_0.8_N_1.2_ confirmed the uniform distribution of V atoms in 2D V_0.2_M_0.8_N_1.2_ (Figure S6). Linear electron energy loss spectroscopy (EELS) was used to determine the alloying mode in V_0.2_Mo_0.8_N_1.2_, Figure 2d. V L edge spectra were collected in three different positions, Figure 2e. There was no V L edge signal in site 1 and site 3, confirming that Mo atoms occupy the site without V alloying on the top. However, site 2 shows the apparent V L_2_ and L_3_ signal, Figure 2f. As shown in Figure 2g, the arithmetic average V L edge peak intensity correlates to the STEM images. These data confirm that the V atoms are alloyed into V_0.2_Mo_0.8_N_1.2_ lattice via the substitution of Mo atoms and underscore the 2D unique solid solution structure.

**Figure 3:**
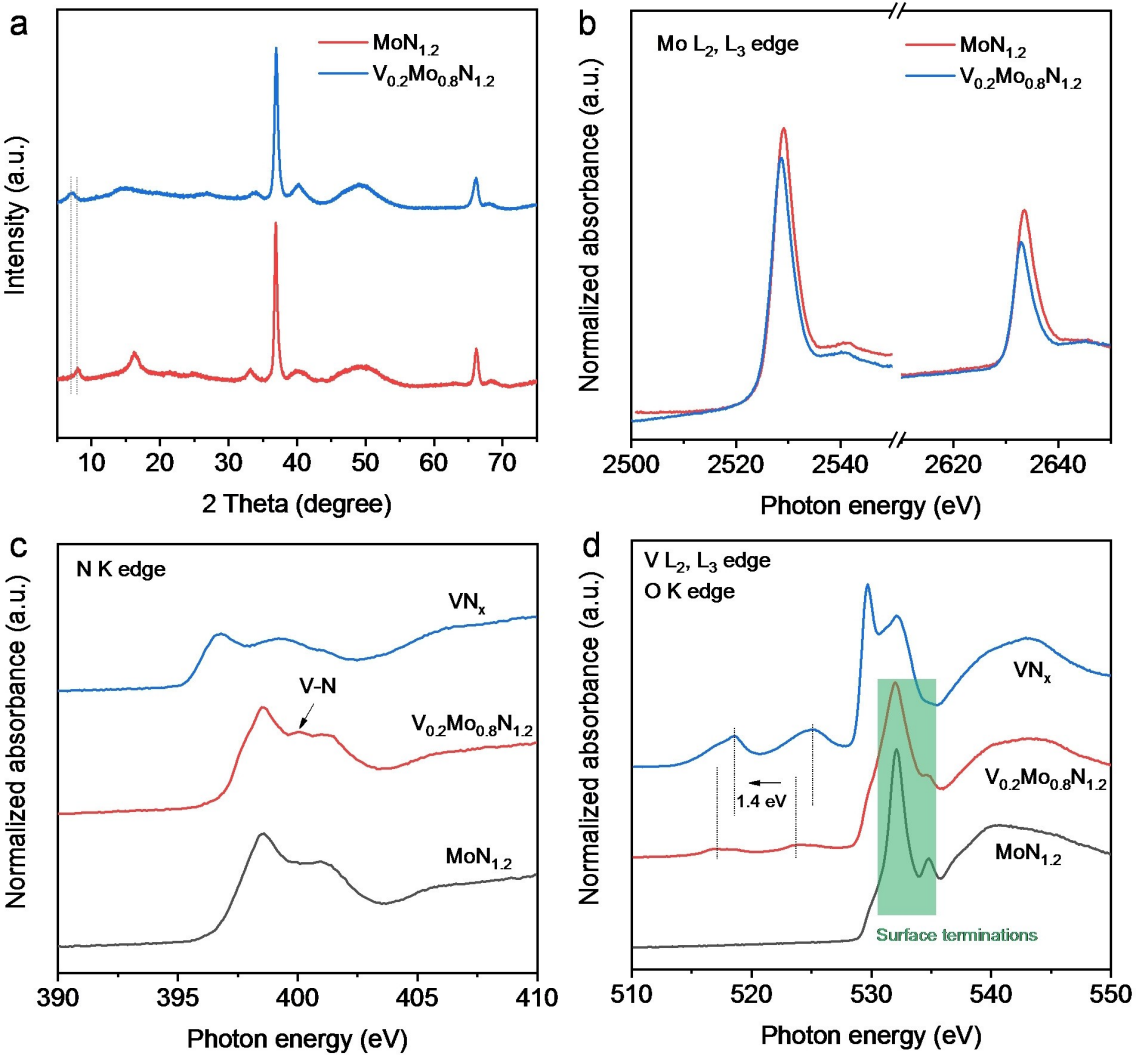
Spectroscopic characterization for V_0.2_Mo_0.8_N_1.2_. a) XRD pattern for V_0.2_Mo_0.8_N_1.2_ and MoN_1.2_. b) Synchrotron‐based Mo L edge XANES spectra for V_0.2_Mo_0.8_N_1.2_ and MoN_1.2_. c) Synchrotron‐based N K edge XANES spectra and d) V L edge and O K edge XANES spectra for V_0.2_Mo_0.8_N_1.2_, VN_*x*_ and MoN_1.2_, respectively.

Crystal structure of V_0.2_Mo_0.8_N_1.2_ was analyzed via X‐ray diffraction (XRD), which confirmed the same crystal structure as for MoN_1.2_, Figure 3a. The peak at 8° confirms the single to few‐layer 2D flakes. The redshift for this peak demonstrates a greater interlayer spacing than for MoN_1.2_, indicating the V alloying changes interlayer interaction. Raman spectra for V_0.2_Mo_0.8_N_1.2_, MoN_1.2_ and VN_*x*_ underscore the effect of solid solution composition on lattice vibrations (Figure S7). The disappearance of several peaks for V_0.2_Mo_0.8_N_1.2_ confirms the change of atomic bonding in the lattice compared with MoN_1.2_ and VN_*x*_. Surface‐sensitive X‐ray photoelectron spectroscopy (XPS) and synchrotron‐based XANES measurements were used to determine the bond structure of V in V_0.2_Mo_0.8_N_1.2_. The XPS survey scan confirmed the similar chemical composition of V_0.2_Mo_0.8_N_1.2_ with MoN_1.2_ (Figure S8a). The Mo/V ratio correlates to the Mo/V ratio in the precursor, confirming that all V atoms in V_2_O_5_ have been dissolved into the 2D lattice. Both Mo L edge XANES spectra, Figure 3b, and Mo 3d XPS spectra (Figure S8b) showed a lower Mo valence state in V_0.2_Mo_0.8_N_1.2_ compared with MoN_1.2_. As is shown in Figure 3b, the Mo L_2_ and L_3_ edge of V_0.2_Mo_0.8_N_1.2_ negatively shifted 0.6 eV. This energy shift confirms that the alloyed V atoms provide electrons to Mo sites and result in a charge redistribution within the 2D matrix. In the N K edge spectra for V_0.2_Mo_0.8_N_1.2_, a new resonance at 400 eV was observed, which attributes to V−N bonding, Figure 3c. The dissolution of V in the lattice is evidenced by the findings from V L edge XANES and XPS. As is shown in Figure 3d, there is an apparent V L edge peak in V_0.2_Mo_0.8_N_1.2_ that confirms the existence of V in the sample. The contrasting shape of V L edge confirms that the V−N bonding in V_0.2_Mo_0.8_N_1.2_ is different from that in VN_*x*_ (Figure S8c). The O K edge spectra for the samples shown in Figure 3d confirm the existence of the O terminations on the surface of 2D nitridene. The invariable O K spectra for V_0.2_Mo_0.8_N_1.2_ and MoN_1.2_ underscore that the alloying did not alter the surface termination. Findings from these characterizations confirm the structure of V_0.2_Mo_0.8_N_1.2_ in which V atoms are alloyed/doped in V_0.2_Mo_0.8_N_1.2_ without phase transformation or separation.

**Figure 4:**
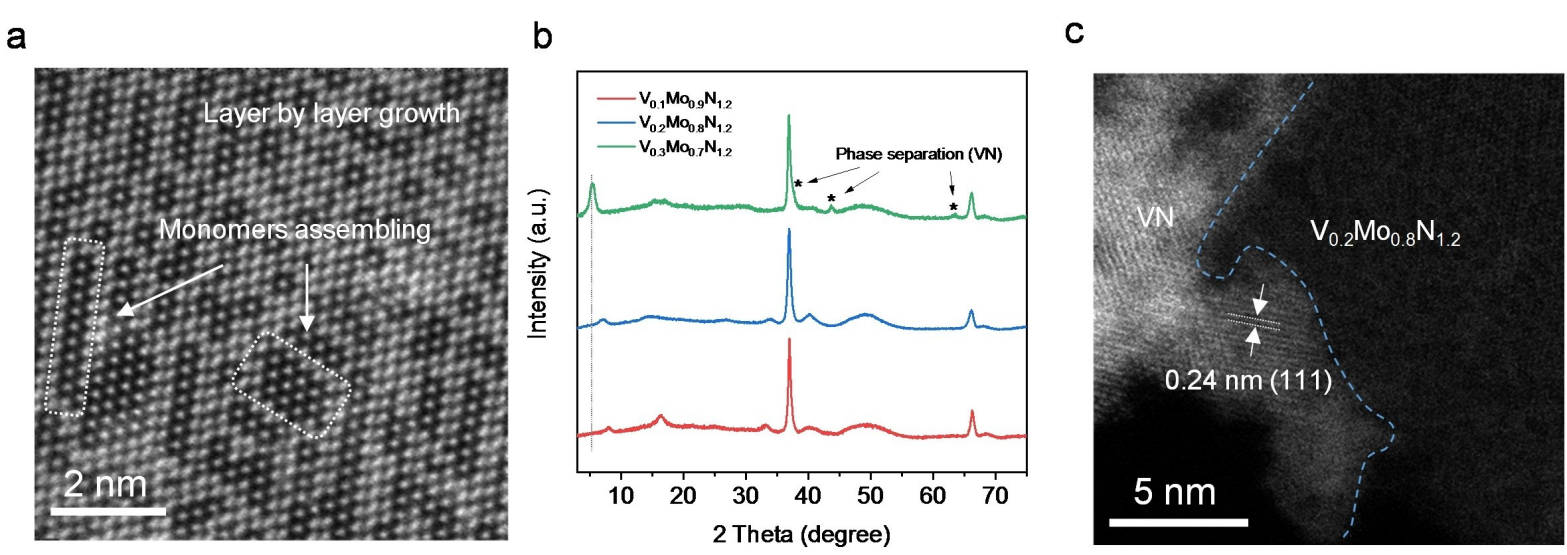
Growth mechanism for 2D V_0.2_Mo_0.8_N_1.2_ solid solution. a) HAADF‐STEM image of V_0.2_Mo_0.8_N_1.2_ at early growth stage. Chain‐shape patterns show monomer assembly. b) XRD patterns for V doped MoN_1.2_ with differing V/Mo ratio. c) HAADF‐STEM image confirming phase separation of VN on V_0.2_Mo_0.8_N_1.2_ surface.

The growth mechanism for the nitridene solid solution was assessed through a series of control experiments. Because the formation of a solid solution needs two isostructural compounds, the lack of 2D layered VN_1.2_ limited the synthesis of V_0.2_Mo_0.8_N_1.2_ using conventional approaches. Therefore, the reaction intermediate in our modified method is the critical factor for analyzing the growth of V_0.2_Mo_0.8_N_1.2_. A control sample was designed by ceasing synthesis at the early stage to permit determination of reaction intermediates. In detail, the tube‐furnace was turned‐off immediately when the temperature reached 650 °C. The tube was removed and cooled at room temperature (25 °C) with protection of NH_3_. The sample was washed using deionized water and characterized via high‐resolution STEM. As is seen in Figure 4a, chain‐shaped patterns were observed on the surface of single‐layer V_0.2_Mo_0.8_N_1.2_. These findings confirm that the growth of nitridene in the molten salt is along the 2D surface by monomer assembling. Additionally, the 2D morphology formation is because of the strong interaction between the in‐plane lattice of nitridene and the Na_2_MoO_4_ salt. The salt interacts with the in‐plane facet of V_0.2_Mo_0.8_N_1.2_ directly and lowers its activation energy.^[10]^ As a result, the growth of V_0.2_Mo_0.8_N_1.2_ along the [110] direction is kinetically favorable and leads to 2D morphology.

An explanation of why the V atoms can be dissolved into the nitridene lattice without 2D layered VN_1.2_ isostructural compounds is as follows: in molten salt, all reactants are melted prior to the growth of TMNs, followed by nitridation of the metal oxide monomers. This leads nitride monomers to assemble on the 2D surface. Molten salt can be considered as a supersaturated system where the polymorph with the lowest nucleation barrier forms initially.^[21]^ In the synthesis of V_0.2_Mo_0.8_N_1.2_, the growth of the metastable MoN_1.2_ polymorph dominates crystallization Kinetics because of the high activity of metal oxide monomers.^[22]^ The [VO_5_] monomer is then linked to MoN_1.2_ atomic chain because of its high concentration. Overall growth kinetics is optimized because of the induction of the alkali metal ions, similar to the growth of other metastable 2D nitridenes.[^19a^, ^20^, ^23^] Based on combined findings, we conclude that the formation of 2D bimetallic nitridene solid solution consists of three steps: 1) the formation of [VO_5_]_pyramidal_ and [MoO_6_]_octahedron_ monomers, 2) nitridation of these seeds [Eq. 1], and; 3) growth of TMN crystals and assembly of [MoN_*x*_] and [VN_*x*_] monomers induced by alkali ions [Eq. 2]. The Na_2_MoO_4_ acts as the catalyst during overall synthesis:

(1) $$\left[\mathrm{MoO}{}_{6}\right]+\left[\mathrm{VO}{}_{5}\right]+\mathrm{NH}{}_{3}\to \left[\mathrm{MoN}{}_{x}\right]{}_{\mathrm{monomer}}+\left[\mathrm{VN}{}_{x}\right]{}_{\mathrm{monomer}}+H{}_{2}O$$

(2) $$\left[\mathrm{MoN}{}_{x}\right]{}_{\mathrm{monomer}}+\left[\mathrm{VN}{}_{x}\right]{}_{\mathrm{monomer}}+\mathrm{Na}{}^{+}+\mathrm{MoO}{}_{4}{}^{2-}\to V{}_{0.2}\mathrm{Mo}{}_{0.8}N{}_{1.2}+\mathrm{Na}{}_{2}\mathrm{MoO}{}_{4}$$

In the next step, the solubility of the V atoms in the nitridene solid solution was assessed because the V atoms might be expected to have limited solubility due to the lack of isostructural 2D layered vanadium nitride.^[12]^ Two control samples with differing V/Mo ratios denoted as V_0.1_Mo_0.9_N_1.2_ and V_0.3_Mo_0.7_N_1.2_ were prepared. The solid solution is well‐maintained when the alloying level is low, Figure 4b (Figure S9a). However, phase separation occurs when the V/Mo ratio reaches 3 : 8, Figure 4b. The VN nanoparticles crystallize from the V_0.2_Mo_0.8_N_1.2_ lattice because of the oversaturated [VO_5_], as is evidenced in the HAADF‐STEM image of Figure 4c and Figure S9b. As shown in Figures S10 and S11, the chemical composition did not change however the Mo valence state decreased with the increase of V concentration, confirming that the V atoms provide electrons to Mo. The reaction rate in different growth directions determines the thickness and lateral size of 2D structured materials.[^10^, ^21b^] With the assistance of alkali metal ions, the growth of 2D V_0.2_Mo_0.8_N_1.2_ proceeds along the in‐plane direction, as shown in Figure 4a. However, with a significant number of V atoms being dissolved into the lattice, the balance of the crystal is broken, leading to phase separation. In consequence, three design principles emerge to guide the synthesis of 2D V_0.2_Mo_0.8_N_1.2_ solid solution: 1) appropriate ratio of alkali metal salt of oxides should be computed carefully to give a stable growth environment, 2) salt must be inactive with ammonia to ensure a catalytic synthesis, and; 3) ratio of foreign atoms, such as V must be less than a specific value to prevent phase separation.

**Figure 5:**
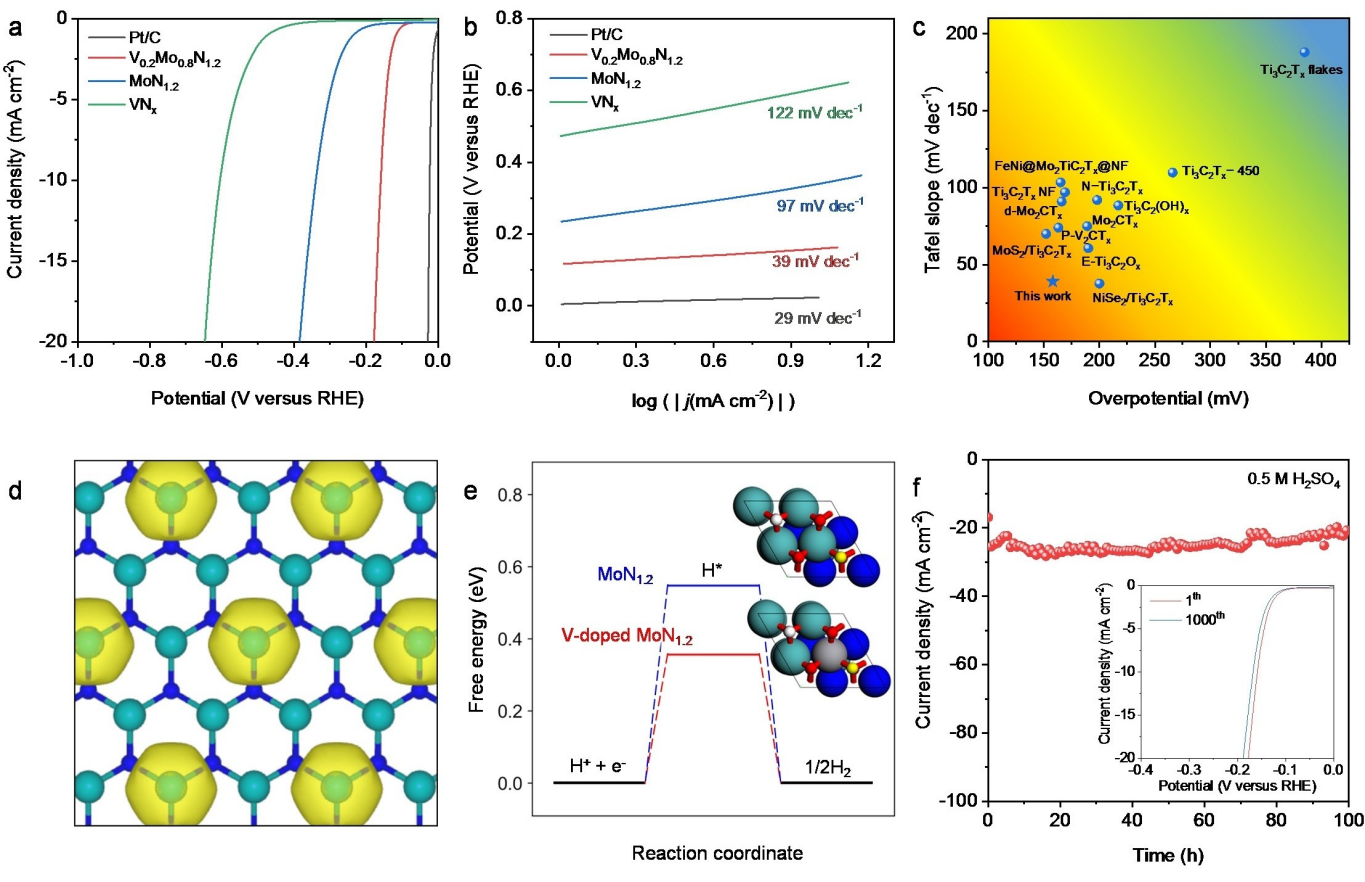
HER performance for 2D V_0.2_Mo_0.8_N_1.2_ solid solution and DFT calculations. a) LSV curves and b) corresponding Tafel plots for selected catalysts in Ar‐saturated 0.5 M H_2_SO_4_ solution. Scan rate=5 mV s^−1^. c) Comparison of η_10_ and Tafel slope for V_0.2_Mo_0.8_N_1.2_ solid solution with reported MXene‐based HER catalysts. d) Differential charge density of V‐doped MoN_1.2_. The yellow contour represents electron accumulation. The isosurface level is set to be 0.05 e Bohr^−3^. e) Free energy diagram for HER on MoN_1.2_ (blue) and V‐doped Mo_5_N_6_ (red). The schematic top view of the corresponding atomic structure is shown as the inset. The adsorption site for *H is marked in yellow. Green spheres: Mo; blue spheres: N; grey spheres: V; red spheres: O; white spheres: H. f) Long‐term stability of V_0.2_Mo_0.8_N_1.2_ solid solution for HER under acid conditions. Inset: LSV curves for V_0.2_Mo_0.8_N_1.2_ solid solution at 1^st^ and 1000^th^ cycle.

Because of the unique electronic structure, we assessed the electrocatalytic HER activity of V_0.2_Mo_0.8_N_1.2_ via electrochemical measurement in 0.5 M H_2_SO_4_. Figure 5a summarizes the linear sweep voltammetry (LSV) curves for different catalysts. V_0.2_Mo_0.8_N_1.2_ exhibited a boosted HER performance, with a η_10_ of 158 mV and a small Tafel slope of 39 mV dec^−1^ (Figure 5b). This is comparable with the Pt/C benchmark of 23 mV and 29 mV dec^−1^. The Tafel slope of V_0.2_Mo_0.8_N_1.2_ is significantly lower than that for MoN_1.2_ and VN_*x*_, demonstrating the optimized electronic structure for HER. In addition, we further investigated the effect of V doping levels on the HER performance of V_0.2_Mo_0.8_N_1.2_. As shown in Figure S12, V_0.2_Mo_0.8_N_1.2_ shows superior HER performance to the control samples of V_0.1_Mo_0.9_N_1.2_ and V_0.3_Mo_0.7_N_1.2_. In addition, the Tafel slope of V_0.2_Mo_0.8_N_1.2_ reduces to 39 mV dec^−1^ compared to that for V_0.1_Mo_0.9_N_1.2_, indicating that the doping process optimizes the Volmer step. However, the phase separation depresses the performance of nitridene solid solution with a Tafel slope of to 50 mV dec^−1^ due to the suboptimal hydrogen adsorption energy of VN nanoparticles. To eliminate the effect of VN_*x*_ and MoN_1.2_ interaction, we prepared a control sample of VN_*x*_‐MoN_1.2_ heterojunction by mixing VN_*x*_ and MoN_1.2_ with the molar ratio of 1 : 4. As shown in Figure S13, the VN_*x*_‐MoN_1.2_ heterojunction shows approximate η_10_ as MoN_1.2_ with a higher Tafel slope of 108 mV dec^−1^, indicating that the phase interaction between MoN_1.2_ and VN_*x*_ is insufficient in facilitating the HER activity. For a comprehensive assessment of the new 2D nitridene solid solution, the η_10_ values and Tafel slope of V_0.2_Mo_0.8_N_1.2_ were compared with other 2D MXene‐based HER catalysts reported in recent studies, Figure 5c (Table S1).[^5c^, ^6a^, ^24^] The value of η_10_ and Tafel slope for V_0.2_Mo_0.8_N_1.2_ are lower than the corresponding values for most of the reported 2D MXene electrocatalysts because of the unique electronic structure optimized by the dissolved V atoms in the solid solution. Additionally, the electrochemically active surface area (ECSA) of the different samples was assessed to gain insight into HER for V_0.2_Mo_0.8_N_1.2_. ECSA is represented by the electrochemical double‐layer capacitance (*C*_dl_) (Figure S14 and S15). Compared with VN_*x*_, V_0.2_Mo_0.8_N_1.2_ has a larger active surface area. However, the ECSA for V_0.2_Mo_0.8_N_1.2_ is similar to MoN_1.2_. Therefore, the boosted HER activity is strongly corroborated by its optimized electronic structure rather than the exposed surface area. In addition, DFT calculations were performed to verify the effect of V alloying on the surface charge distribution and HER performance. The average Bader charge of Mo atoms on the surface of V‐doped MoN_1.2_ is +0.56, lower than that on the pristine MoN_1.2_ surface of +0.65, confirming the charge transfer between V and Mo. Differential charge density diagram shows that the alloying of V increases the electron density on the catalyst surface (Figure 5d). Reaction free energy diagram further verifies that such an effect of electron accumulation caused by V alloying further enhances the binding strength of the HER reaction intermediate *H at the active sites on the catalyst surface, resulting in higher reactivity (Figure 5e and S16). DFT calculations of charge transfer and free energy match well with the experiments. Thus, we conclude that V alloying weakens the hydrogen bonding energy of the V−N−Mo site and facilitates overall HER.

In addition to activity, the primary practical parameter for MXene‐based HER electrocatalyst is stability.^[5c]^ In acid electrolytes, V_0.2_Mo_0.8_N_1.2_ exhibits excellent electrocatalytic stability over 100 h (Figure 5f), better than control samples and most MXenes (Figure S17). This electrocatalytic stability demonstrates that the material is active and corrosion resistant in acidic environment. Post‐reaction characterizations were further conducted to determine the phase‐structure properties for V_0.2_Mo_0.8_N_1.2_ after HER. As shown in Figure S18, both the morphology and atom arrangement for V_0.2_Mo_0.8_N_1.2_ remain unchanged, demonstrating the stable feature of the crystal structure of nitridene solid solution. In addition, the Mo L edge, V L edge, and O K edge XANES spectra had no visible variation (Figure S19), confirming a stable chemical state for different elements in the 2D lattice. The highly significant stability originates from the unique metal‐nitrogen bonding in 2D nitridene solid solution and the inherent anti‐corrosive property of 2D TMNs.

## Conclusion

In summary, a new MXene analogue, 2D layered V_0.2_Mo_0.8_N_1.2_ solid solution, was synthesized for high‐rate hydrogen production. Compared with conventional MXenes, the V_0.2_Mo_0.8_N_1.2_ exhibited both significant HER activity and long‐term electrocatalytic stability. Furthermore, XANES spectra and DFT calculation confirmed that V_0.2_Mo_0.8_N_1.2_ has a modified electronic structure, leading to the electron transfer from V to Mo sites for fast protons coupling to produce hydrogen. Findings offer a quantitative strategy for development of MXene analogues for high‐rate hydrogen production and clean energy conversions and will be of immediate interest and benefit to the design of new and low‐cost 2D materials based on earth‐abundant transition metals.

## Conflict of interest

The authors declare no conflict of interest.

1

## Supporting information

As a service to our authors and readers, this journal provides supporting information supplied by the authors. Such materials are peer reviewed and may be re‐organized for online delivery, but are not copy‐edited or typeset. Technical support issues arising from supporting information (other than missing files) should be addressed to the authors.

Supporting Information

## Data Availability

The data that support the findings of this study are available from the corresponding author upon reasonable request.
