# Supplementary material for: MXene Analogue: A 2D Nitridene Solid Solution for High‐Rate Hydrogen Production
Source: Angew Chem Int Ed Engl. 2022 May 3;61(27):e202203850. doi: 10.1002/anie.202203850 (PMC9322295; doi:10.1002/anie.202203850)
Supplement: Supplementary file 1 — Supporting Information [file ANIE-61-0-s001.pdf]

Angewandte Chemie
Eine Zeitschrift der Gesellschaft Deutscher Chemiker

Supporting Information

MXene Analogue: A 2D Nitridene Solid Solution for High-Rate Hydrogen Production

*H. Jin, H. Yu, H. Li, K. Davey, T. Song, U. Paik, S.-Z. Qiao**

Experimental Procedures

Chemicals: MoO_3 (99.97 %), V_2O_5 (99.95 %) and $Na_2MoO_4 \cdot 2H_2O$ (≥ 99.5 %) were purchased from Sigma-Aldrich without further purification, and 5 % NH_3/Ar gas from BOC, Australia.

Synthesis of 2D layered $MoN_{1.2}$ nanosheet. 2D $MoN_{1.2}$ nanosheets were synthesized *via* three-steps, namely, 1) 1.44 g of MoO_3 powder and 2.42 g of $Na_2MoO_4 \cdot 2H_2O$ powder (mole ratio 1:1) was mixed *via* ball-milling in a planetary micro-mill (Fritsch Pulverisette 7 premium line) with a rotation speed 400 rpm for 20 min (two cycles), 2) 100 mg of this mix was evenly positioned in a porcelain-boat (to be noted is that if the thickness of the accumulation is too large (> 1 mm) the reaction will be incomplete because of formation of a molten-salt shield) and 2D $MoN_{1.2}$ produced by annealing the as-prepared precursor at 650 °C for 5 h with a ramp rate of 1 °C min^{-1} under 5% NH_3/Ar atmosphere, and; 3) product was washed *via* ultra-sonication in deionized water to remove salt.

Synthesis of 2D layered $V_xM_{1-x}N_{1.2}$ solid solution. V_2O_5 and MoO_3 were mixed together with mole ratio 1:9, 2:8 and 3:7. This V_2O_5/MoO_3 mix was blended with $Na_2MoO_4 \cdot 2H_2O$ (mole ratio 1:1) *via* ball milling. 2D $V_xM_{1-x}N_{1.2}$ solid solution was produced by annealing the as-prepared precursor at 650 °C for 5 h at a ramp rate 1 °C min^{-1} under 5% NH_3/Ar atmosphere. Product was washed *via* ultra-sonication in deionized water to exfoliate the 2D nanosheets and remove salt.

Characterization. X-Ray Powder Diffraction (XRD) data were collected on a Rigaku MiniFlex 600 X-Ray Diffractometer. The aberration-corrected transmission electron microscope images, high-angle annular dark-field imaging, and EDS and EELS mapping, were taken on a FEI Titan Themis 80-200 operating at 200 kV. X-ray photoelectron spectroscopy (XPS) analyses were carried out under ultra-high vacuum on a Kratos Axis Ultra with a Delay Line Detector photoelectron spectrometer using an aluminum monochromatic X-ray source.

Synchrotron-based XANES measurements were carried out on the soft X-ray spectroscopy beamline at the Australian Synchrotron equipped with a hemispherical electron analyzer and a microchannel plate detector that permits simultaneous recording of the total and partial, electron yield. Raw XANES data were calibrated and normalized using Igor Pro 8 software.

Raman measurements. Raman spectroscopy was obtained using a confocal Raman microscope (Horiba LabRAM HR Evolution) with a 100X objective (Olympus). The laser wavelength was 532 nm.

Electrochemical measurements. 5 mg of catalyst e.g. $V_{0.2}Mo_{0.8}N_{1.2}$ was dispersed in 900 μL of deionized water to which 100 μL 1 wt% of Nafion/water was added, and sonicated for 20 min. Because the catalysts are electrically conductive, carbon black was not added. 10 μL of catalyst dispersion (5 mg

mL^{-1}) was dropped onto a 5 mm glassy-carbon rotating-disk electrode serving as working electrode. The reference electrode was Ag/AgCl in saturated KCl, and the counter electrode a graphite rod. All potentials were referenced to the reversible hydrogen electrode (RHE) by adding, (0.197 + 0.059 × pH) V, and polarization curves corrected for iR compensation within the cell. A flow of Ar was maintained over the 0.5 M H_2SO_4 electrolyte during experiment to eliminate dissolved oxygen. The working electrode was rotated at 1,600 rpm to remove hydrogen which was formed on the catalyst surface.

Computational details. DFT calculations were performed with the package QuantumESPRESSO (QE),[1] with the van der Waals-corrected BEEF-vdW exchange correlation functional.[2] The two-dimensional structural model of $MoN_{1.2}$ (001) from our previous work was used,[3] with a (2 × 2) unit-cell in size of (5.72 × 5.72) sin(60°) Å and the separation by a vacuum region of 20 Å. All structures were fully relaxed until residual forces fell below 0.03 eV/Å using a density and wave function cut-off of 6000 and 600 eV, respectively. A *k*-point mesh of (4 × 4 × 1) and (12 × 12 × 1) was used for the structural relaxation and charge density calculations, respectively.

In comparison to the pristine $MoN_{1.2}$, one of the 4 Mo atoms on the surface layer in each unit-cell was replaced with a V atom to simulate the V doping of around 25% which is close to the experimental ratio of 20%. Similar to the MXene materials, the surface configuration covered by *OH groups was used for the calculation of *H adsorption under electrochemical conditions. The side views of the bare and *OH-covered V-doped $MoN_{1.2}$ structures are shown in Figure S16. The adsorption free enegy of *H (G_H) was calculated as: $G_H = E_H + ZPE + \int C_p dT - TS$, where ZPE is the zero-point energy, $\int C_p dT$ is the enthalpic correction, TS is the entropic correction, E_H is the adsorption energy of *H: $E_H = E_{surf+H} - E_{surf} - 1/2\mu(H_2)$, $\mu(H_2)$ is the chemical potential of H_2 molecule.

Supplementary Figures

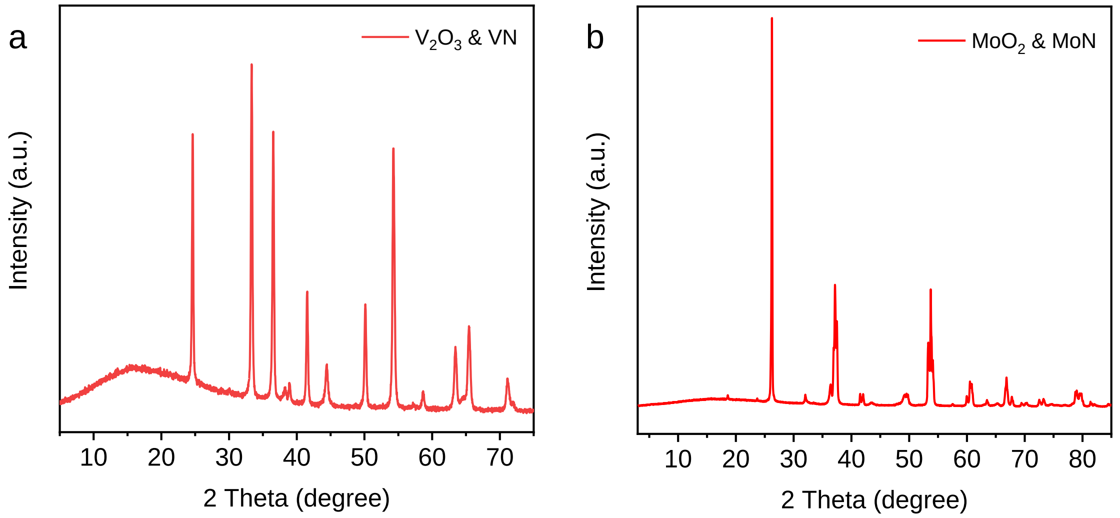

Figure S1. XRD patterns for samples from direct nitridation of V_2O_5 and WO_3 powder without addition of Na_2MoO_4.

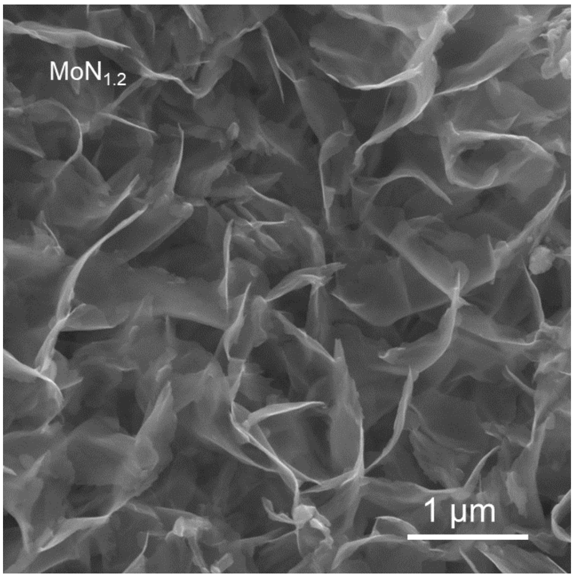

Figure S2. SEM image of $MoN_{1.2}$ nanosheets.

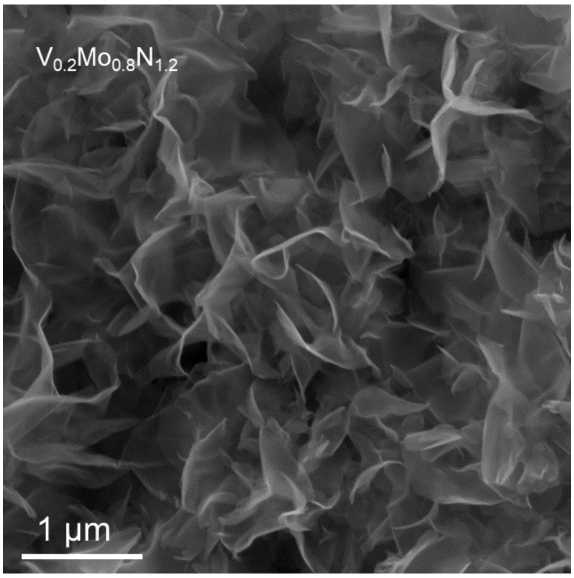

Figure S3. SEM image of 2D $V_{0.2}M_{0.8}N_{1.2}$ solid solution nanosheets.

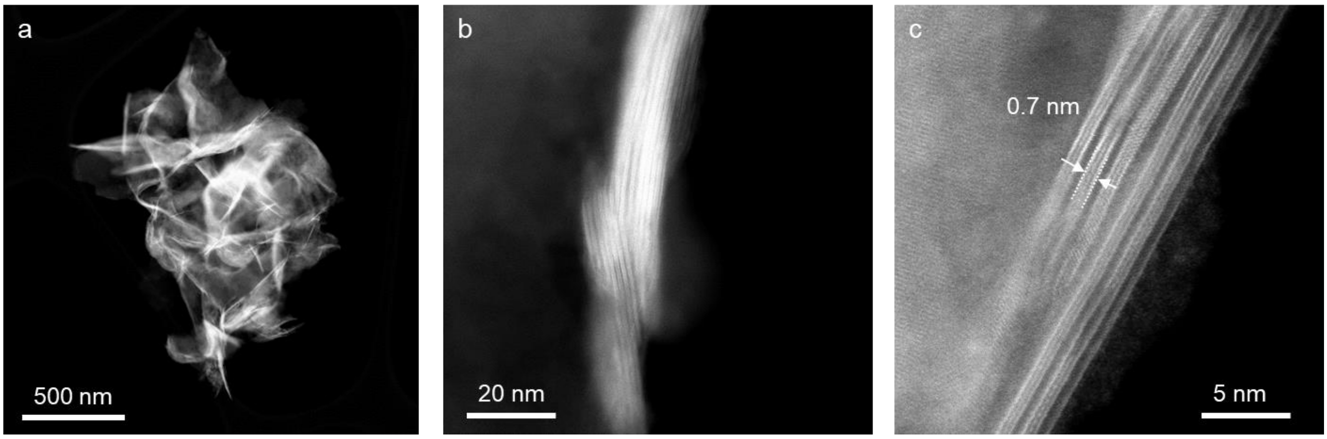

Figure S4. (a) Low-resolution STEM image of $MoN_{1.2}$ confirming 2D morphology. (b) Low-resolution cross-sectional HAADF-STEM image of a few-layer $MoN_{1.2}$ nanosheet showing the laminated structure. (c) High-resolution cross-sectional HAADF-STEM image of a few-layer $MoN_{1.2}$ confirming single-layer thickness.

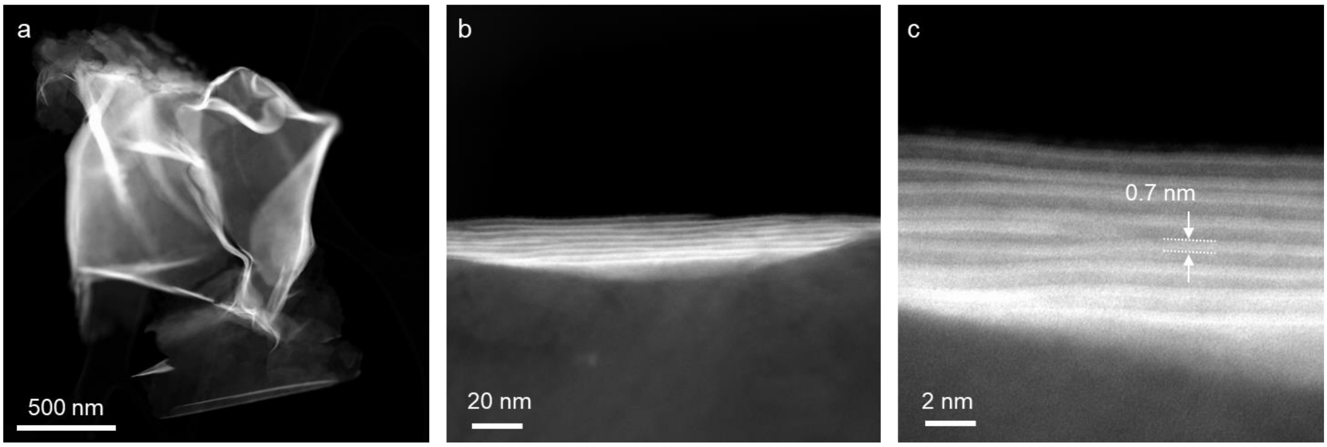

Figure S5. (a) Low-resolution STEM image of $V_{0.2}M_{0.8}N_{1.2}$ confirming 2D morphology (similar to $MoN_{1.2}$). (b) Low-resolution cross-sectional HAADF-STEM image of a few-layer $V_{0.2}M_{0.8}N_{1.2}$ in which the laminated structure can be seen. (c) High-resolution cross-sectional HAADF-STEM image of a few-layer $V_{0.2}M_{0.8}N_{1.2}$ confirming single-layer thickness (similar to $MoN_{1.2}$).

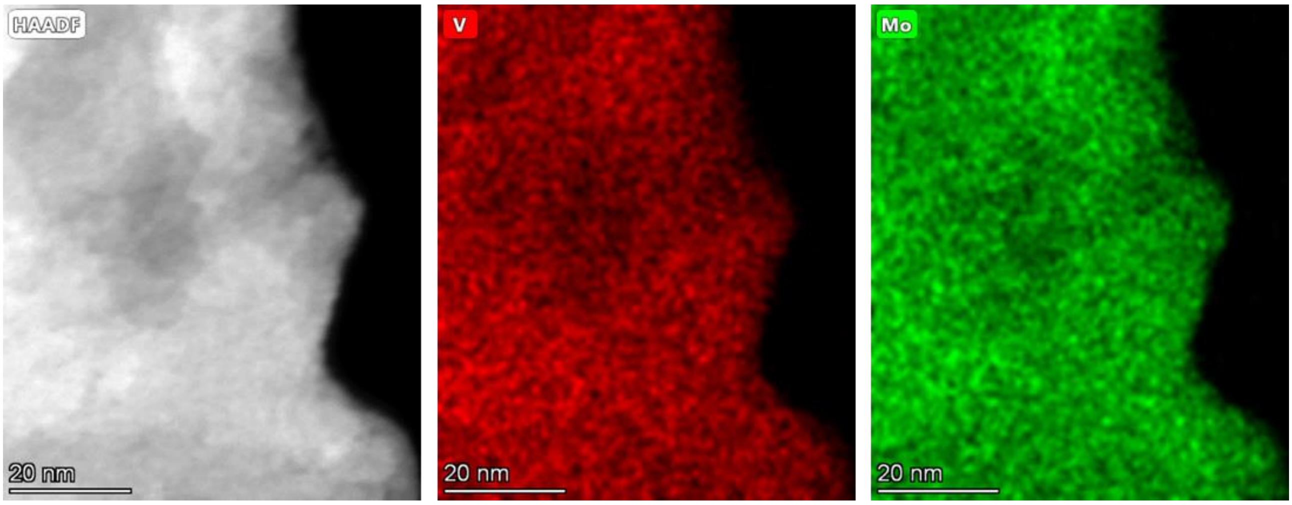

Figure S6. Large-area EDS mapping for $V_{0.2}M_{0.8}N_{1.2}$ that confirms uniform distribution of V atoms in 2D $V_{0.2}M_{0.8}N_{1.2}$ solid solution.

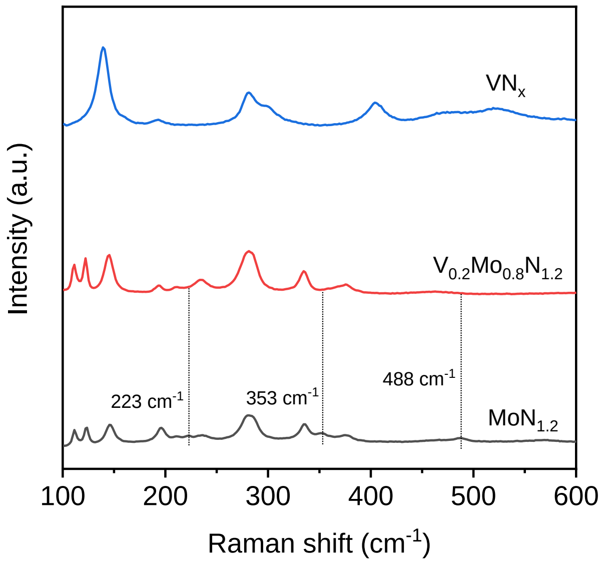

Figure S7. Raman spectra for VN_x, $V_{0.2}Mo_{0.8}N_{1.2}$ and $MoN_{1.2}$ under ambient conditions (25 ^{o}C).

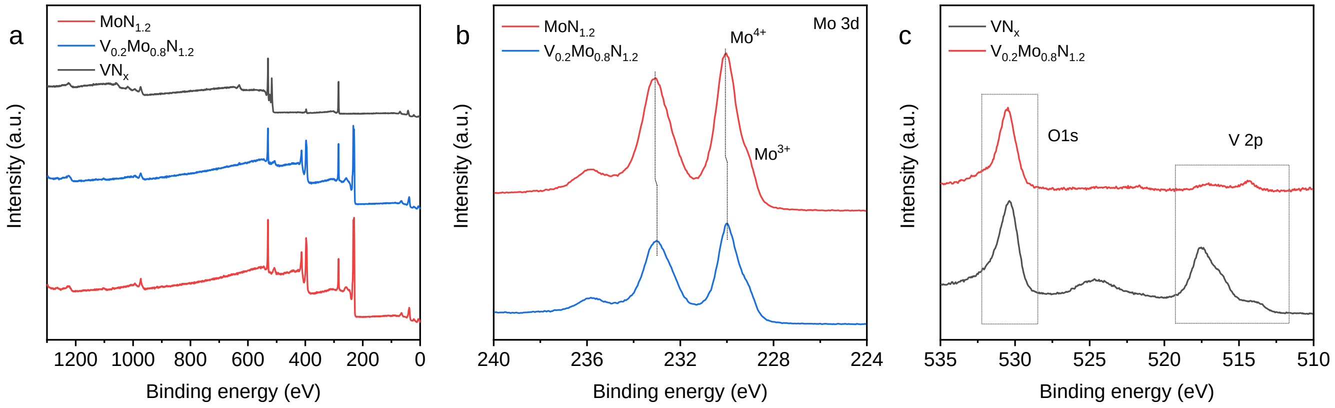

Figure S8. (a) XPS survey scan for, respectively, VN_x, $V_{0.2}Mo_{0.8}N_{1.2}$ and $MoN_{1.2}$. (c) High-resolution Mo 3d XPS spectra for $V_{0.2}Mo_{0.8}N_{1.2}$ and $MoN_{1.2}$. (b) High-resolution V 2p and O 1s XPS spectra for $V_{0.2}Mo_{0.8}N_{1.2}$ and VN_x.

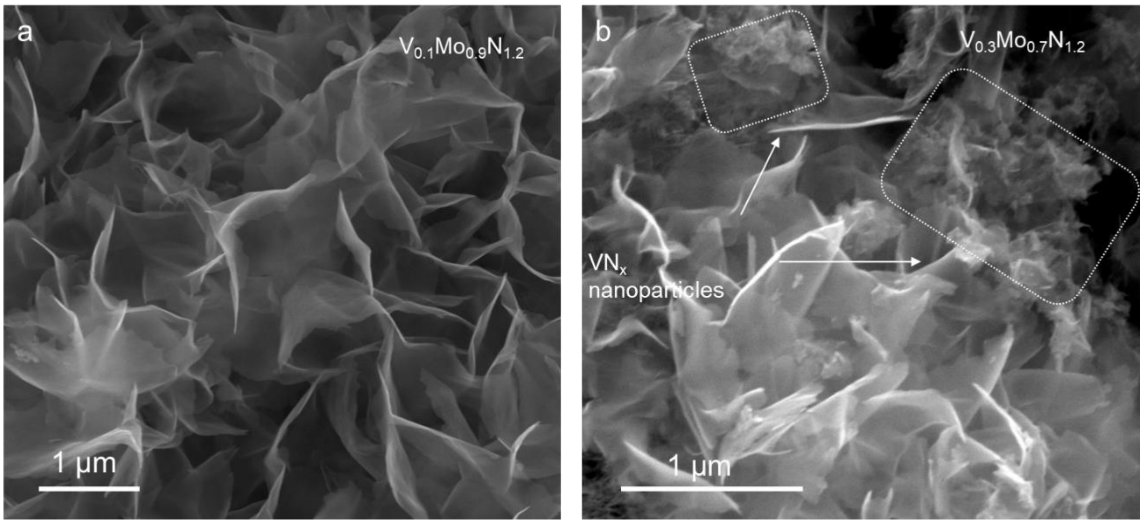

Figure S9. (a) SEM image of $V_{0.1}M_{0.9}N_{1.2}$ nanosheet. (b) SEM image of $V_{0.3}M_{0.7}N_{1.2}$ nanosheet. The highlighted small nanoparticles are the phased-separated VN_x because of saturation of V atoms in the solid solution.

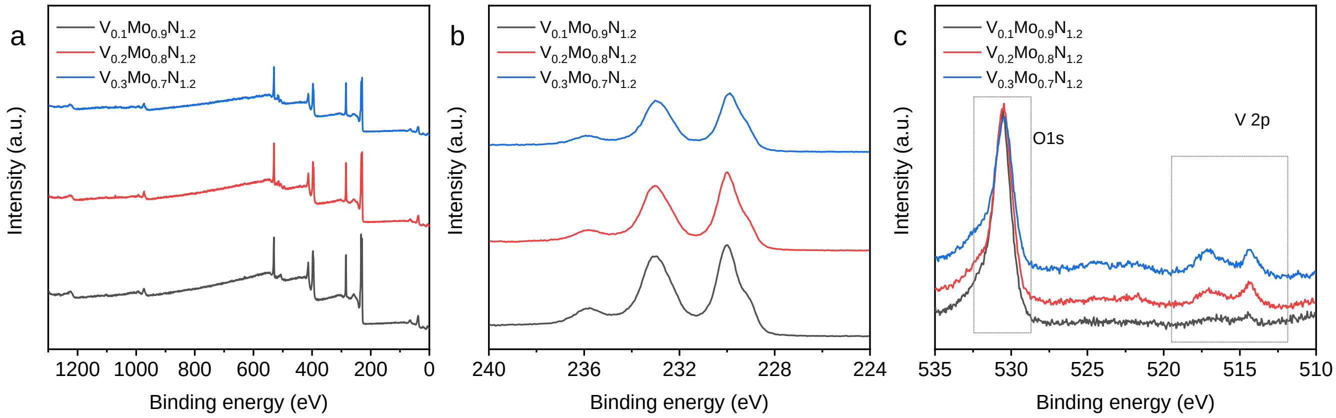

Figure S10. (a) XPS survey scan, (b) High-resolution Mo 3d XPS spectra, and (c) High-resolution V 2p and O 1s XPS spectra for $V_{0.1}Mo_{0.9}N_{1.2}$, $V_{0.2}Mo_{0.8}N_{1.2}$ and $V_{0.3}Mo_{0.7}N_{1.2}$.

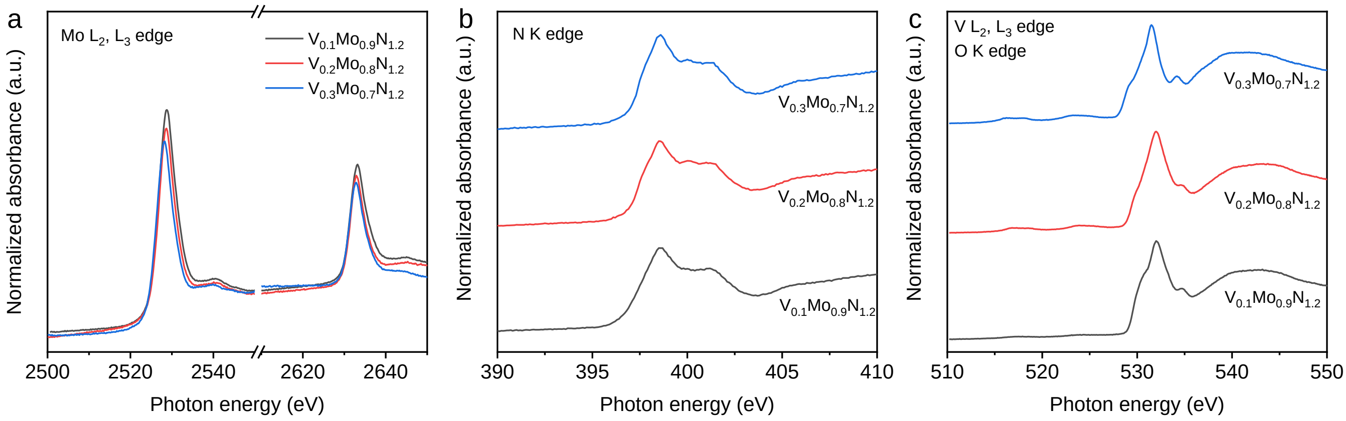

Figure S11. Synchrotron-based characterizations for $V_{0.1}Mo_{0.9}N_{1.2}$, $V_{0.2}Mo_{0.8}N_{1.2}$ and $V_{0.3}Mo_{0.7}N_{1.2}$. (a) Mo L edge, (b) N K edge and (c) V L edge and O K edge XANES spectra.

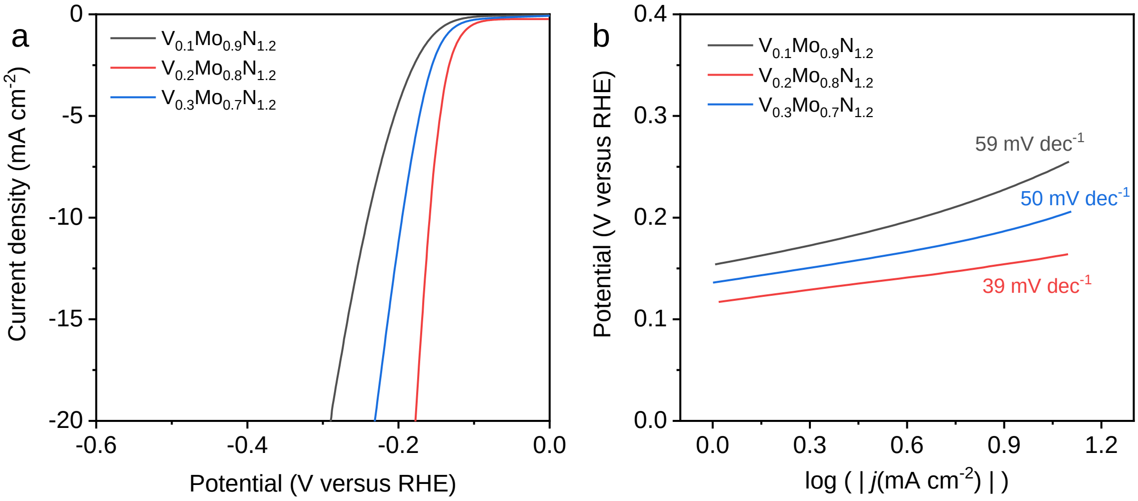

Figure S12. (a) LSV curves and (b) Tafel plots for $V_{0.1}Mo_{0.9}N_{1.2}$, $V_{0.2}Mo_{0.8}N_{1.2}$ and $V_{0.3}Mo_{0.7}N_{1.2}$ in Ar-saturated 0.5 M H_2SO_4 solution.

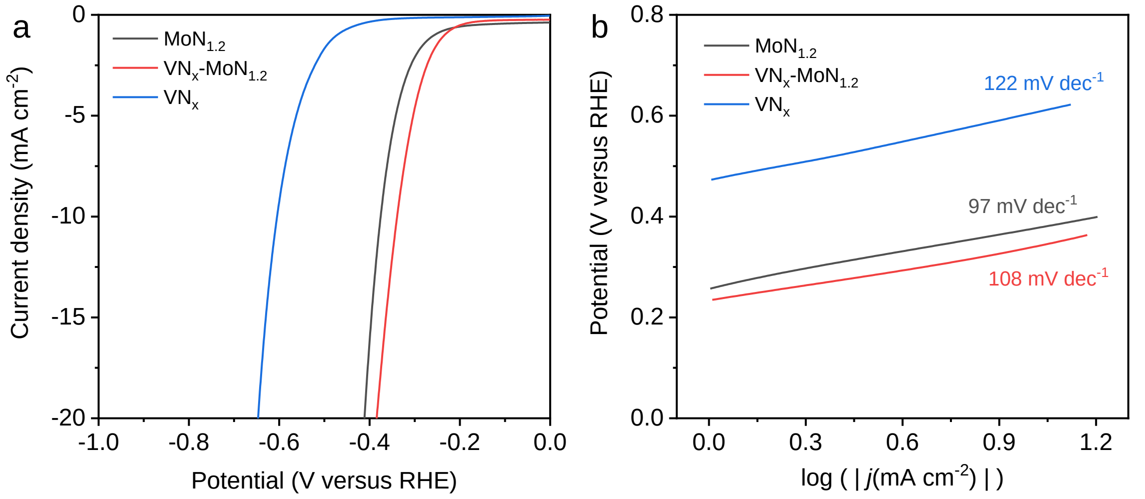

Figure S13. (a) LSV curves and (b) Tafel plots for $MoN_{1.2}$, VN_x and VN_x-$MoN_{1.2}$ in Ar-saturated 0.5 M H_2SO_4 solution.

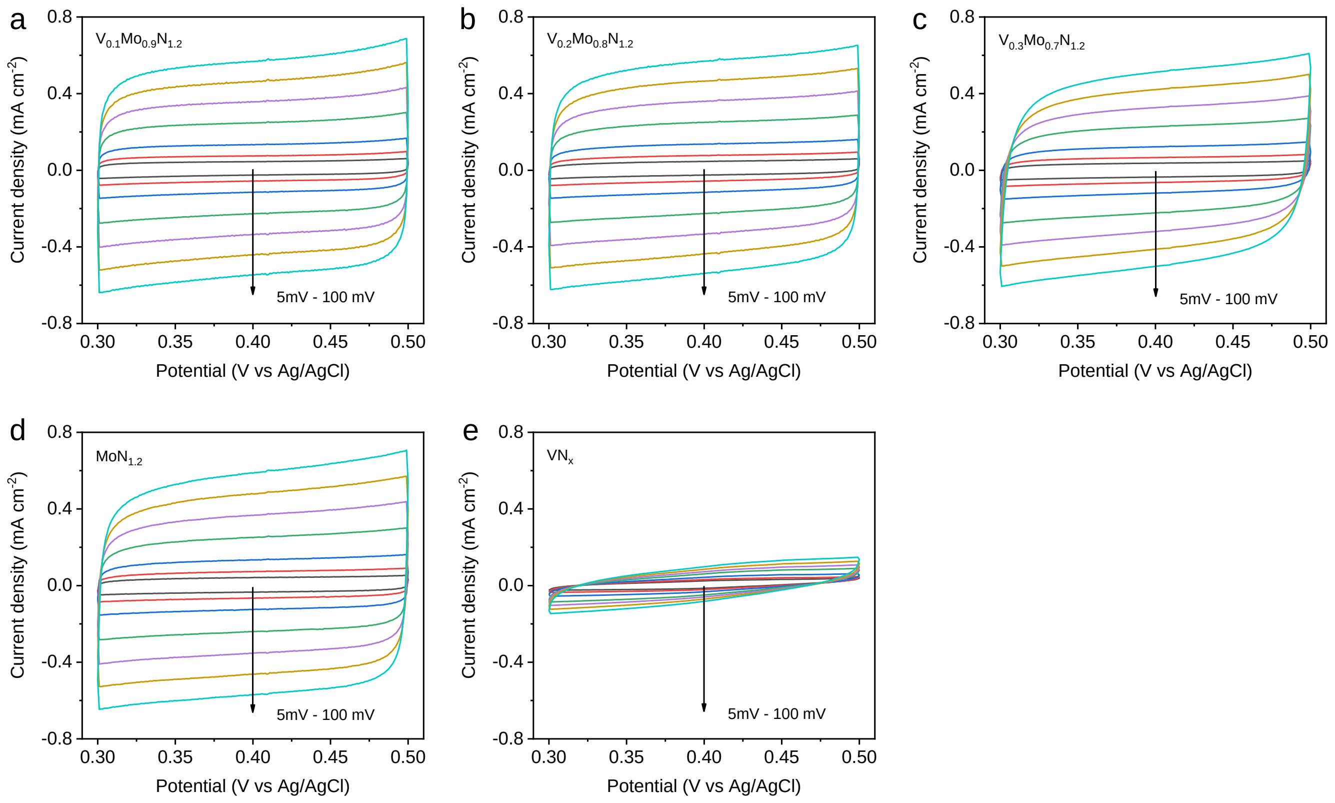

Figure S14. CV curves for samples for ECSA evaluation.

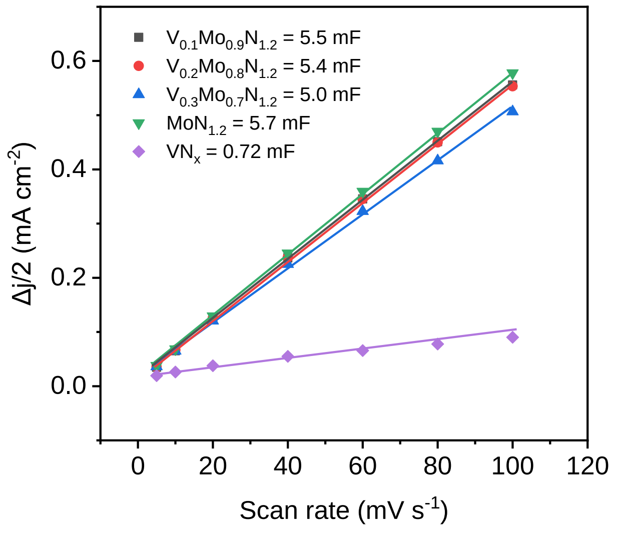

Figure S15. Double-layer capacitance (C_{dl}) for catalysts as represented by curve slope.

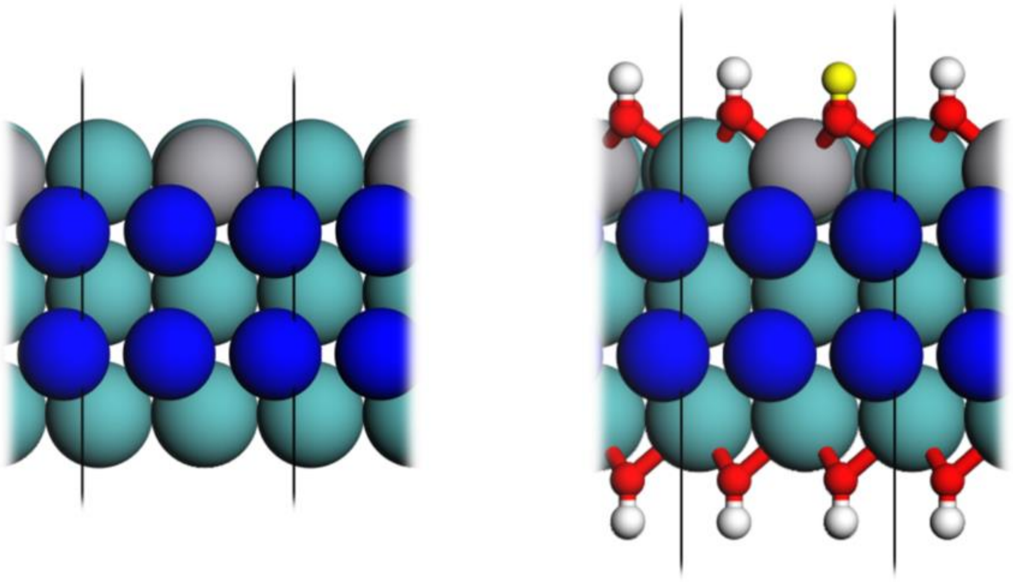

Figure S16. Computational models for bare (left) and *OH-covered V-doped (right) $MoN_{1.2}$ structures. The adsorption site for *H is marked in yellow. Green spheres: Mo; blue spheres: N; grey spheres: V; red spheres: O; white spheres: H.

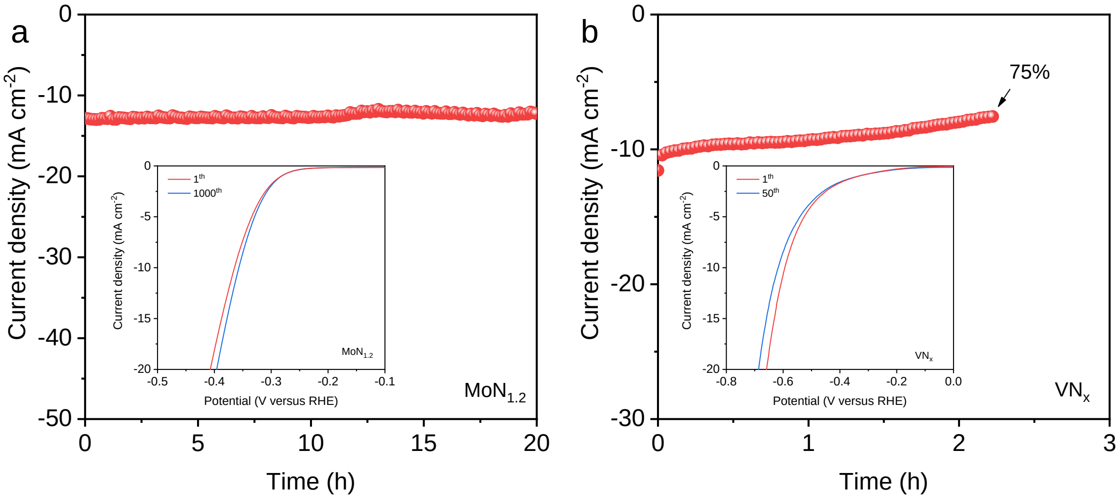

Figure S17. Chronoamperometry test for (a) $MoN_{1.2}$ and (b) VN_x. Inset: LSV curves for $MoN_{1.2}$ at 1st and 1000th, and VN_x at 1st and 50th, respectively.

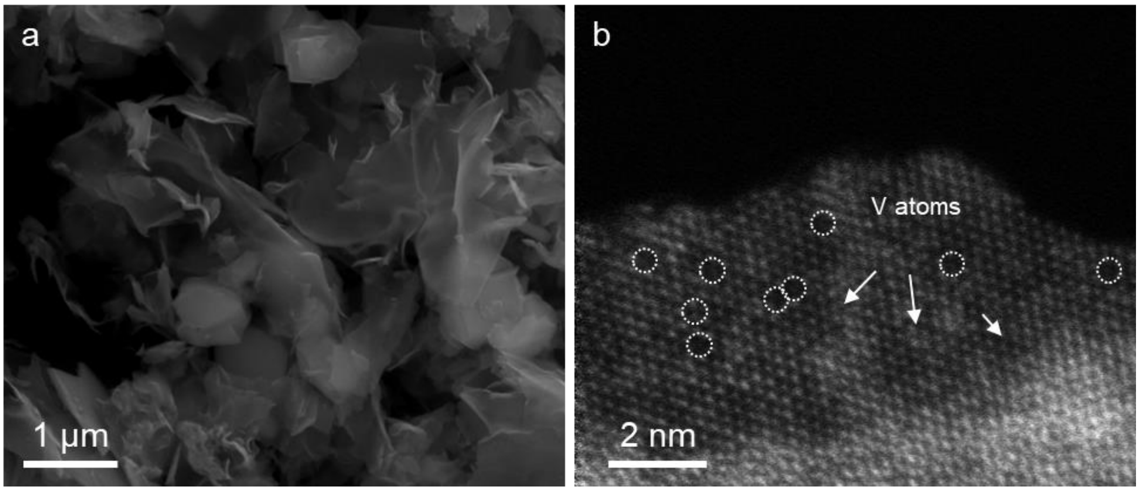

Figure S18. (a) SEM image and (b) high-resolution HAADF-STEM image of 2D $V_{0.2}M_{0.8}N_{1.2}$ following HER stability test. V atoms are highlighted by white-color open-circles.

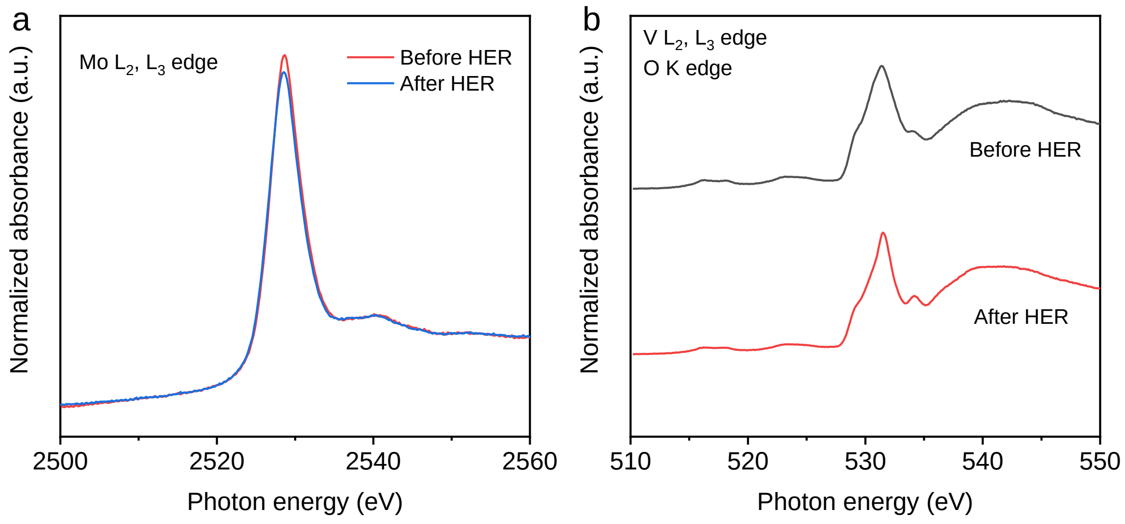

Figure S19. Synchrotron-based (a) Mo L edge, and (b) V L edge and O K edge XANES spectra $V_{0.2}Mo_{0.8}N_{1.2}$ following HER stability test.

Table S1. Comparative summary of HER activity of 2D $V_{0.2}Mo_{0.8}N_{1.2}$ with selected 2D MXene-based electrocatalysts under acid condition.

Sample	η_{10} (mV)	Tafel slope (mV dec^{-1})	Electrolyte	Ref.
$V_{0.2}Mo_{0.8}N_{1.2}$	158	39	0.5 M H_2SO_4	This work
P-V_2CT_x	163	74	0.5 M H_2SO_4	[4]
E-$Ti_3C_2O_x$	190	60.7	0.5 M H_2SO_4	[5]
$Ti_3C_2(OH)_x$	217	88.5	0.5 M H_2SO_4	[5]
$Ti_3C_2T_x$-450	266	109.8	0.5 M H_2SO_4	[5]
$Ti_3C_2T_x$ nanofibers	169	97	0.5 M H_2SO_4	[6]
$Ti_3C_2T_x$ flakes	385	188	0.5 M H_2SO_4	[6]
N−$Ti_3C_2T_x$	198	92	0.5 M H_2SO_4	[7]
$MoS_2/Ti_3C_2T_x$	166	91	0.5 M H_2SO_4	[8]
$NiSe_2/Ti_3C_2T_x$	200	37.7	0.5 M H_2SO_4	[9]
$MoS_2/Ti_3C_2T_x$ nanoroll	152	70	0.5 M H_2SO_4	[10]
FeNi@$Mo_2TiC_2T_x$@NF	165	103.46	0.5 M H_2SO_4	[11]
d-Mo_2CT_x	189	70	0.5 M H_2SO_4	[12]

References

[1] a) P. Giannozzi, et al., *J. Phys.: Condens. Matter* **2009**, *21*, 395502; b) P. Giannozzi, et al., *J. Phys.: Condens. Matter* **2017**, *29*, 465901.

[2] J. Wellendorff, K. T. Lundgaard, A. Møgelhøj, V. Petzold, D. D. Landis, J. K. Nørskov, T. Bligaard, K. W. Jacobsen, *Phys. Rev. B* **2012**, *85*, 235149.

[3] C. Ye, H. Jin, J. Shan, Y. Jiao, H. Li, Q. Gu, K. Davey, H. Wang, S.-Z. Qiao, *Nat. Commun.* **2021**, *12*, 7195.

[4] Y. Yoon, A. P. Tiwari, M. Choi, T. G. Novak, W. Song, H. Chang, T. Zyung, S. S. Lee, S. Jeon, K.-S. An, *Adv. Funct. Mater.* **2019**, *29*, 1903443.

[5] Y. Jiang, T. Sun, X. Xie, W. Jiang, J. Li, B. Tian, C. Su, *ChemSusChem* **2019**, *12*, 1368-1373.

[6] W. Yuan, L. Cheng, Y. An, H. Wu, N. Yao, X. Fan, X. Guo, *ACS Sustain. Chem. Eng.* **2018**, *6*, 8976-8982.

[7] T. A. Le, Q. V. Bui, N. Q. Tran, Y. Cho, Y. Hong, Y. Kawazoe, H. Lee, *ACS Sustain. Chem. Eng.* **2019**, *7*, 16879-16888.

[8] K. R. G. Lim, A. D. Handoko, L. R. Johnson, X. Meng, M. Lin, G. S. Subramanian, B. Anasori, Y. Gogotsi, A. Vojvodic, Z. W. Seh, *ACS Nano* **2020**, *14*, 16140-16155.

[9] H. Jiang, Z. Wang, Q. Yang, L. Tan, L. Dong, M. Dong, *Nano-Micro Letters* **2019**, *11*, 31.

[10] J. Liu, Y. Liu, D. Xu, Y. Zhu, W. Peng, Y. Li, F. Zhang, X. Fan, *Appl. Catal., B* **2019**, *241*, 89-94.

[11] J. Wang, P. He, Y. Shen, L. Dai, Z. Li, Y. Wu, C. An, *Nano Res.* **2021**, *14*, 3474-3481.

[12] Z. W. Seh, K. D. Fredrickson, B. Anasori, J. Kibsgaard, A. L. Strickler, M. R. Lukatskaya, Y. Gogotsi, T. F. Jaramillo, A. Vojvodic, *ACS Energy Lett.* **2016**, *1*, 589-594.

Author Contributions

S-Z.Q. conceived and supervised the work. H.J. designed the research. H.J. and H.Y. conducted the synthesis, characterizations and electrochemical measurements. H.L. designed and performed DFT calculations. T.S. and U.P. assisted analyses of findings. H.J., H.Y., H.L., K.D. and S-Z.Q. wrote the manuscript. H.J. and H.Y. contributed equally to this work.
